# Design of an Integrated Micro-Viscometer for Monitoring Engine Oil

**DOI:** 10.3390/s22145157

**Published:** 2022-07-09

**Authors:** Roufaida Bensalem, Animesh Saha Shovan, Juan Morency Trudel, Hani H. Tawfik, Karim Allidina, Mohannad Y. Elsayed, Mounir Boukadoum, Mourad N. El-Gamal

**Affiliations:** 1 Electrical and Computer Engineering Department, McGill University, Montreal, QC H3A 0E9, Canada; rofaida.bensalem@mail.mcgill.ca (R.B.); animesh.saha@mail.mcgill.ca (A.S.S.); juan.morencytrudel@mail.mcgill.ca (J.M.T.); 2 MEMS Vision International Inc., Montreal, QC H4P 2R9, Canada; hani.tawfik@mems-vision.com (H.H.T.); karim.allidina@mems-vision.com (K.A.); mohannad.elsayed@mems-vision.com (M.Y.E.); 3 Department of Computer Engineering, Université du Québec à Montréal, Montreal, QC H2X 3Y7, Canada; boukadoum.mounir@uqam.ca

**Keywords:** ASIC, CMUT, oil condition, MEMS, viscometer, charge pump, sensors

## Abstract

This paper proposes a novel integrated micro-viscometer for engine-oil monitoring. The final solution consists of a capacitive micromachined ultrasonic transducer (CMUT) and an application-specific integrated circuit (ASIC). The CMUT is used to generate and capture acoustic waves while immersed in engine oil. The low power transceiver ASIC is interfaced with the CMUT structure for actuation and reception. An integrated charge pump boosts the supply voltage from 3.3 to 22 V to generate the DC polarization voltage of the CMUT. The receiver has a power consumption of 72 µW with an input-referred noise current of 3.2$\frac{pA}{\sqrt{Hz}}$ and a bandwidth of 7 MHz. The CMUT array occupies an area of 3.5 × 1 mm, whereas the ASIC has a chip area of 1 × 1 mm. The system was tested using engine oils of different types and ages at different temperatures. Measurement results show a significant frequency shift due to the dynamic viscosity change that occurs as oil ages. A shift of −1.9 kHz/cP was measured, which corresponds to a shift of 33 Hz/mile. This work paves the way for high accuracy-integrated solutions for oil condition monitoring and is expected to play a significant role in a more economic and environmentally friendly usage of oil.

## 1. Introduction

Motor oil is a viscous liquid used as a lubricant for protecting engines from wearing. In internal combustion engines, oil is prone to strains caused by a variety of factors such as ambient conditions, operating parameters, and fuel quality. The rate of its degradation depends on a combination of these elements. To prevent engine failure, the oil must be replaced before it loses its protective properties. It is primarily changed based on time/mileage intervals and not on a need basis, which means that oil that is still useful is being wasted. One million gallons of water is contaminated by used oil from one oil change. This is equivalent to a year’s supply of freshwater for 50 American citizens [1]. To avoid the associated environmental and economic impact of unnecessary oil changes, periodic monitoring of the oil’s physical and chemical state is required. This will reliably determine the optimum oil change interval [2]. One method for determining oil’s quality is to measure its viscosity, which is defined as its resistance to flow. This is one of the most important properties used to analyze the characteristics of any fluid. In the automotive industry, the viscosity of the engine oil must be kept within a certain range to ensure its effectiveness. Therefore, viscosity is a primary indicator that can be used to evaluate the condition of an engine oil. Conventional engine oil monitoring methods rely on assessing the oil conditions indirectly through monitoring the usage of the car itself, e.g., the mileage from the last oil change, the number of cold start-ups, the oil temperature, and/or the engine speed [3]. Advances in research have led to the development of sensors to directly monitor the oil quality and to improve measurement accuracy [4]. For large-scale stationary engines, a comprehensive test approach is used where oil samples are tested in the laboratory at regular intervals to obtain the optimal times for oil changes. However, this method is costly and requires a time delay between taking the sample and obtaining the test results [2]. To enable widespread use, a fast and accurate in situ method of monitoring the oil condition is needed.

There are three categories of in situ oil condition sensors commonly used in the industry [5,6]. The first category consists of dielectric sensors, which measure the dielectric constant of the oil to assess its ability to resist an electric field from forming in it. The second category consists of conductivity sensors [7], which report the conductivity of the oil and how it is affected by the presence of soot particles. The third category consists of electromagnetic viscosity sensors [8], which determine oil viscosity by measuring the time it takes for a piston to move through the oil.

Some studies have focused on alternative approaches for monitoring in situ oil conditions by immersing sensors in the oil. These include a cylinder capacitive sensor used to monitor changes in the dielectric constant of the oil [9] and photonic sensors based on the light scattering phenomenon to determine the engine oil quality from its color [10,11]. Ultrasonic techniques using microcantilevers have also been used to monitor engine oil conditions [12,13]. Microcantilevers tend to have limited measurement ranges [14]. In comparison, using capacitive micromachined ultrasonic transducers (CMUTs) to perform the ultrasonic measurements enables higher quality factor devices and simplicity of functionalization, due to the structure of CMUT, which has a flat membrane surface

## 2. CMUT and ASIC Design Methodology

**Figure 1:**
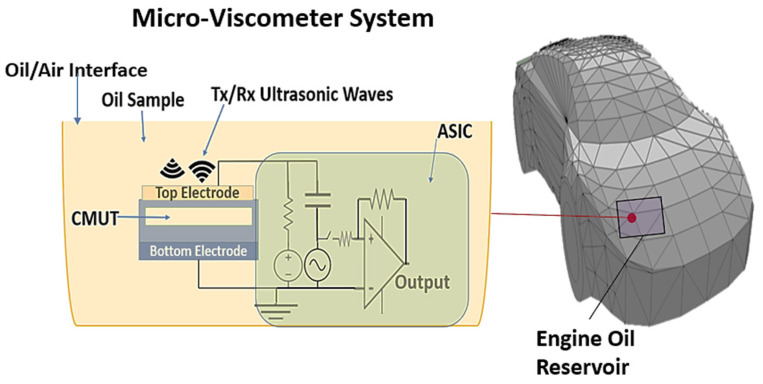
Overall micro-viscometer system design.

The two main building blocks of this system are the CMUT and the ASIC. The basic operating principle is to immerse the CMUT module in the oil and then excite it using a transmitter on the ASIC. This causes the CMUT to emit an acoustic signal, which is reflected by the interface between the engine oil and the air, as shown in Figure 1. The reflected signal is amplified by the receiver on the ASIC and is processed to obtain two parameters: (1) the frequency shift which is processed to determine the viscosity of the oil, and (2) the time-of-flight (ToF) of the acoustic signal which is used to calculate the level of the fluid as a secondary output. This work focuses on using the CMUT technology to implement the ultrasonic transducer due to its unique advantages compared to piezoelectric transducers. CMUTs tend to have a wider bandwidth, which leads to better axial resolution when used in pulse-echo mode [15,16]. In addition, a CMUT array can be seamlessly integrated with front-end electronics, which simplifies the integration and packaging process [17]. The principle of operation and design of the CMUT used in this work are described in the next two sub-sections.

### 2.1. Theoretical Model

**Figure 2:**
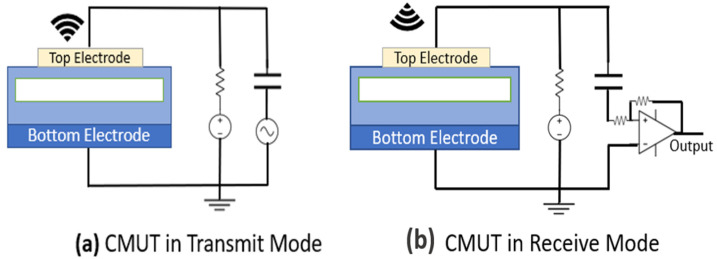
CMUT operation during (**a**) transmission and (**b**) reception.

A CMUT is a MEMS structure that consists of two electrodes facing each other, one fixed and the second movable. The two electrodes are separated by a cavity that is either filled with air or a vacuum. When an alternating electrostatic force is applied between these two electrodes, the movable electrode vibrates and generates an acoustic wave, as shown in Figure 2.

A mechanical analysis of the structure shows that the fundamental vibrational frequency of a circular membrane with an imposed boundary condition in a vacuum $\left(f_{vacuum}\right)$ is affected by the material properties of the membrane [15]. Given a fundamental resonant frequency in a vacuum $\left(f_{vacuum}\right)$ of a CMUT membrane, its vibrating energy will be influenced when immersed in the fluid. This energy will be partially dissipated by acoustic radiation and viscous damping, the amount of which will be dependent on the viscosity of the fluid. As a result, its resonant frequency ($f_{fluid}$) will be changed in proportion to:

(1) $$f_{fluid}=\frac{f_{vacuum}}{\sqrt{1+\beta }}\;,\;$$

where β is the added virtual mass introduced by the fluid. The lubricating oils used in automotive combustion engines have relatively high dynamic viscosities (>10 cP). Therefore, the model of Kozlovsky [18] can be used to determine the added virtual mass β by the fluid as follows:

(2) $$\beta =0.6538\frac{\rho_{fluid}\times a}{\rho_{membrane}\times h}\left(1+1.082\xi \right)\;,$$

where *h* is the thickness of the membrane, a is the radius of the membrane, $\rho_{membrane}$ is the density of the membrane material, and $\rho_{fluid}$ is the density of the fluid. The energy dissipation (ξ) related to the fluid dynamic viscosity (η) can be calculated as

(3) $$\xi =\sqrt{\frac{\eta }{\rho_{fluid}\times 2\pi f_{fluid}\times a^{2}}}\;.$$

when a vibrating plate comes into contact with a fluid, the normal frequency of the plate is reduced—a condition caused by the fluid’s induced vibration. These mediated motions are caused by parasitic waves. The vibrations can also be expressed as a surface of fluid adjacent to the plate that vibrates with it. The plate vibrates as if its mass is increased by the mass of the virtual layer of vibrating fluid, and consequently, its natural frequency decreases. This phenomenon is known as the added-virtual-mass effect [18]. The frequency response of CMUT will be affected by the viscosity of the fluid in contact with it due to the resultant damping effect and the added virtual mass. The ultrasonic signal generated from the CMUT will also be changed accordingly. This change in the CMUT resonant frequency is detected by inspecting the Fourier transformation of the pulse-echo time domain ultrasound signal transmitted in the fluid. From the value of the resonance frequency shift, information about the viscosity of the surrounding medium can be extracted [15].

### 2.2. Structure and Fabrication

**Figure 3:**
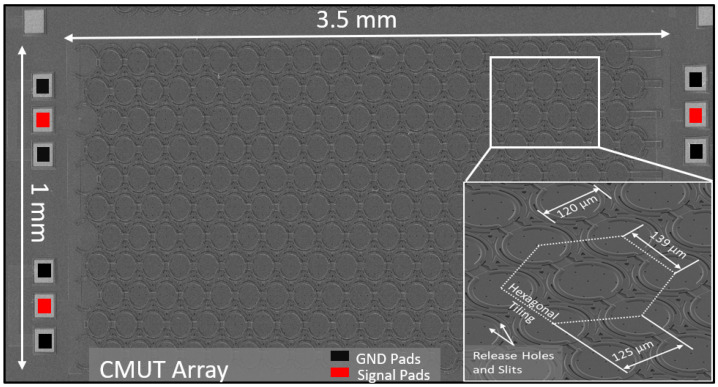
SEM image of the fabricated CMUT array with a hexagonal element distribution.

The transducer implemented in this work is an array of CMUTs realized in the PolyMUMPs technology [19]. The reduced gap structure used is designed for immersion applications, namely, immersion in engine oil for viscosity measurements. Compared to conventional architecture, the reduced-gap structure requires a bias voltage that is almost four times lower for the same magnitude of vibration [19]. The array was fabricated in a 1 × 3.5 mm configuration (Figure 3). It is made of circular cells with a hexagonal element distribution to maximize the output pressure generated by the relatively small CMUT aperture.

The PolyMUMPs process uses a silicon wafer that includes a 600 nm low-stress silicon nitride (SiN) layer that was deposited using low-pressure chemical vapor deposition (LPCVD) as the substrate [20]. The fabrication process starts by depositing a PolySi-0 layer using LPCVD and patterning it with reactive ion etching (RIE). Next, a phosphosilicate glass (PSG-1) sacrificial layer is deposited and patterned. Typically, PolySi-0 serves as the lower electrode in a conventional design, but in this device, PolySi-1 acts as the lower electrode for the CMUT membrane cell. A 0.75 µm PSG-2 sacrificial layer is deposited and patterned to define the anchors of the CMUT vibrating membrane. Next, the CMUT membrane is formed using the PolySi-2 deposition and patterning. A thin layer of gold is deposited and patterned using lift-off to form the pads. The CMUT is then released in a solution of 49% hydrofluoric acid (HF) before going through a critical point drying procedure. To safely immerse the fabricated device in engine oil, a Parylene-C film is used to coat the device. Parylene-C is well known for its impermeability. Therefore, it is an ideal material to block moisture and residues from damaging the device once immersed in oil.

### 2.3. Analog Front End (AFE)—Transceiver ASIC

**Figure 4:**
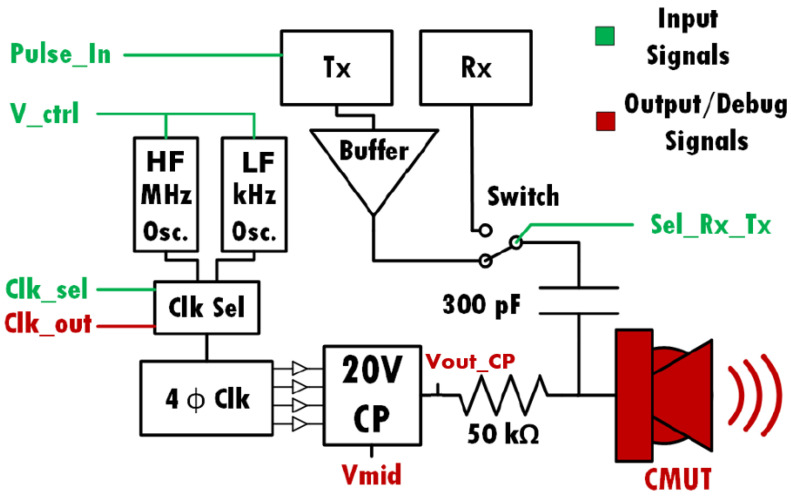
System diagram of the transmitter and charge pump interface.

The block diagram of the electronic interface system is shown in Figure 4. It is composed of three primary circuits: a charge pump, a pulse generator, and a receiver. The charge pump boosts the DC supply to a high voltage for biasing the CMUT device. The pulse generator circuit actuates the biased CMUT device by sending out electrical pulses through a switch connecting it to the CMUT. The CMUT converts the pulses to acoustic signals that propagate through the medium. Once the membrane has sent out an acoustic pulse, the switch connects the CMUT to the receiver circuit. Any movement of the membrane (such as when an acoustic wave impacts with the CMUT) generates an alternating current (AC) which is amplified and detected by the receiver chain. The pulse-echo electronics are typically only active during the small time frame required to send and receive an acoustic pulse to minimize power consumption.

#### 2.3.1. Transmitter

The transmitter is a digital pulse generator that sends a buffered 3.3 V pulse to excite the CMUT. This relatively low pulse voltage can be used because the CMUT is already actuated by the high voltage output of around 22 V DC from the charge pump, which reduces the voltage of the pulse needed to generate an acoustic signal.

#### 2.3.2. Charge Pump

To generate the high voltage CMUT bias from a standard 3.3 V supply, a DC-to-DC converter is needed. Two classes of DC-to-DC voltage amplifiers are commonly used: switching converters [21] and charge pump circuits [22]. A switching converter uses an inductor, a capacitor, and a switching device to achieve high power and low loss. This topology is less attractive for IC design since inductors occupy a significant chip area. In addition, a high-power signal is not needed for this application, because the load is capacitive in nature. Conversely, charge pumps (CP) use a switch-based architecture to charge up capacitors, which means that only transistors and capacitors are needed. This type of circuit is more favorable for IC integration in terms of area. However, generating a 22 V output using standard CMOS technology is still challenging, since the transistors and junctions cannot handle this level of voltage directly without suffering damage. The signals in the system diagram shown in Figure 4 that pertain to the transmitter and charge pump are as follows: The *Pulse_In* signal is sent to the transmitter from a microcontroller when the CMUT needs to transmit an acoustic wave, and the transmitter buffers this signal to properly drive the CMUT. The *Sel_Tx_Rx* signal controls the switch that connects either the transmitter or receiver to the CMUT, and the *Clk_sel* signal determines the frequency of the clock that is sent to the charge pump. The ~19.5 MHz clock is used during the charging phase to build up the bias voltage on the CMUT relatively quickly. When the final voltage has been reached, the high-frequency oscillator is turned off, and the ~25.67 kHz oscillator is used to save power while maintaining the voltage. This low frequency can be used to maintain the charge pump since the CMUT is a purely capacitive load and has an expected leakage current below 10 nA. The *V_ctrl* signal is a debug signal used for fine-tuning the oscillator frequency. The system also outputs three additional debug signals: the charge pump clock signal *Clk_out* and the middle and final charge pump voltages *Vmid* and *Vout_CP*.

**Figure 5:**
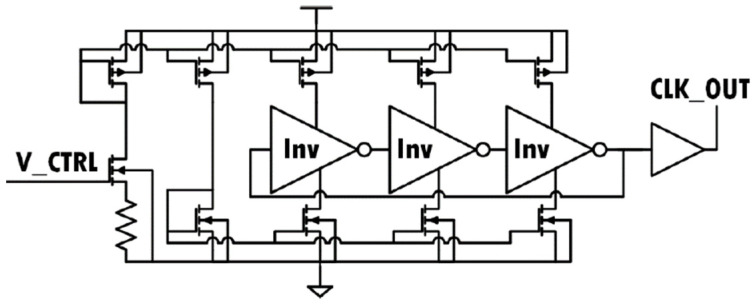
Linear variable frequency oscillator design for an all PMOS.

The clock selection is achieved through simple transmission gates controlled by the external signal *clk_sel* (Figure 4). The clock signal for the charge pump is generated by an on-chip ring oscillator based on the topology shown in Figure 5. This circuit uses a current starving design to limit the current in the inverters of the ring oscillator and therefore achieves the desired frequency [23]. The current starving transistors are 280 nm wide × 120 nm long. However, the transistors that take in *V_ctrl* and the transistors used in the inverters are scaled up to increase the capacitance and hence reduce the frequency.

**Figure 6:**
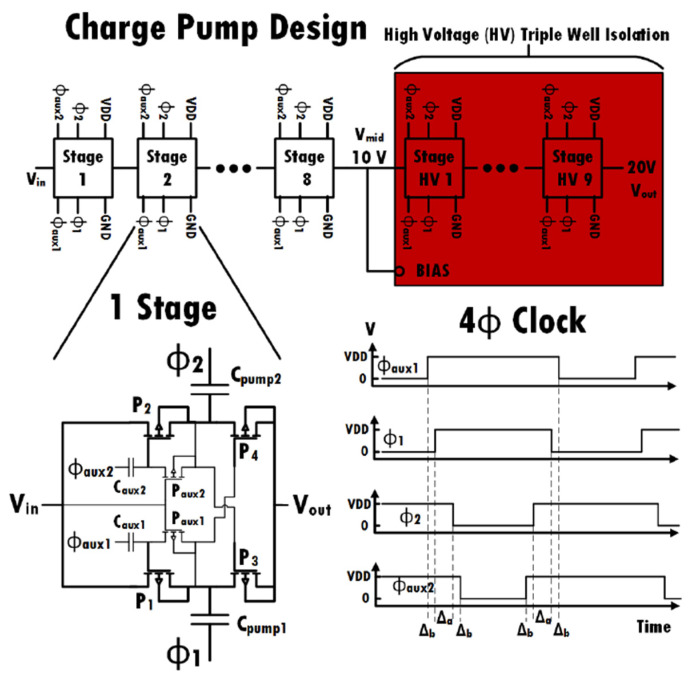
All PMOS charge pump design using high voltage isolation.

**Figure 7:**
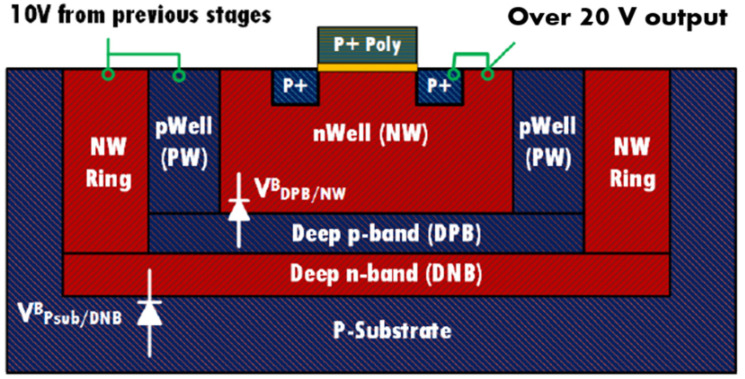
Triple-well isolation.

An all PMOS charge pump design was used in this work. It is a PMOS variant of the Pelliconi CP [24], with an auxiliary circuit to drive the voltage of the first transistors P1 and P2 shown in Figure 6. This allows the circuit to avoid the voltage drop across these transistors, which would normally be of one PMOS threshold voltage. Seventeen (17) stages were used to achieve the final charge pump output voltage (22 V). For stages that handle voltages higher than 10 V, all the PMOS transistors were isolated with a triple-well isolation as shown in Figure 7. This configuration was used to avoid the breakdown of the PN junctions within the transistors when subject to higher voltages. The voltage generated from half of the charge pump stages was used to bias the p-Well (PW) and n-Well (NW) to a middle voltage around 10 V. A four-phase clock circuit is used to drive the designed CP from the ring oscillator.

**Figure 8:**
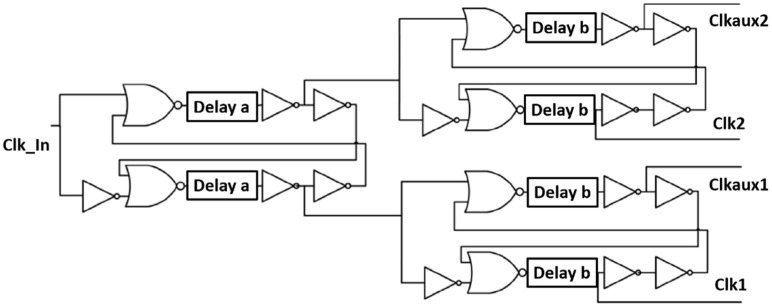
Phase generation circuit.

The circuit that generates the four-phase clock is shown in Figure 8. This digital circuit uses inverters as delay elements in an SR latch topology to create non-overlapping clocks that are separated in phase by 90°. From simulations, it was found that the optimal delays for Δa and Δb were around 100 and 800 ps, respectively. These delays are generated by the appropriate number of inverters in the delay block.

#### 2.3.3. Receiver

**Figure 9:**
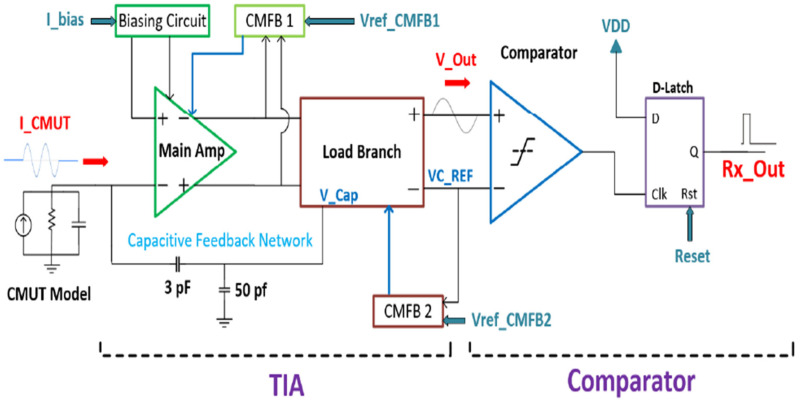
Analog receiver chain.

Figure 9 presents the overall block level diagram of the receiver chain. The input to the receiver chain is an attenuated AC signal represented by I_CMUT, and the output of the system is a pulse that comes out of the D-Latch referred to as Rx Out. The transimpedance amplifier (TIA) is composed of the main amplifier, the capacitive feedback network, and the load branch. The output of the TIA is a voltage signal from the load branch, which is processed by the comparator to determine if a valid echo has been received from the CMUT. The load branch also sets the reference voltage and the threshold voltage for the comparator. The comparator has a buffer stage built into it, which allows for a full range of operation, i.e., 0 to VDD, and the output of the buffer acts as the trigger for the D-Latch. The D-Latch passes a logic one at the output as soon as the output signal from the comparator goes high. The logic one is maintained for roughly 2 µS; thus, it can be processed by an external controller, after which a built-in reset signal sets the output to logic zero before the arrival of the next input signal. Since the CMUT is a single-ended device, the main amplifier is a pseudo-differential telescopic cascode amplifier with its associated bias circuitry. Two main advantages of this differential amplifier, compared to its single-ended counterpart, are the higher output swing and the avoidance of mirror poles, thus resulting in a higher closed-loop speed.

**Figure 10:**
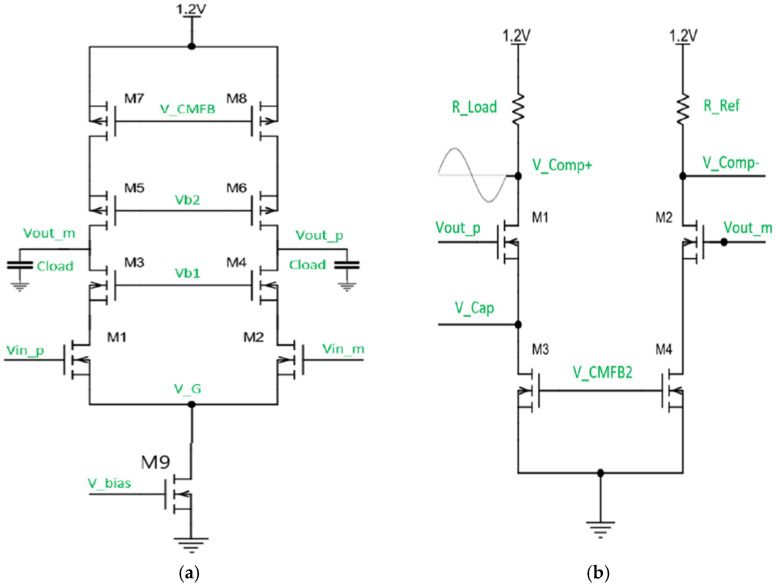
(**a**) Telescopic operational transconductance amplifier. (**b**) Load branch.

In this design of the telescopic amplifier, the output swing is limited by the five cascode transistors. However, to increase the output swing even higher, transistor M9 (Figure 10a) was designed to be near the sub-threshold region. This means that a minimal amount of overdrive voltage is required for the transistor to be in saturation, hence increasing the output swing. In addition, the output is set to mid-rail, which is 600 mV, and this is set by a common mode feedback amplifier to maximize the output swing.

A high gain differential amplifier requires a common mode feedback circuit to set the output common-mode voltage to a known value, as differential feedback cannot define the common-mode level. This is needed to ensure proper operation in the presence of inherent random mismatches between the top and bottom current sources in the amplifier circuit after fabrication. The first common-mode feedback circuit (CMFB1) sets the common-mode voltage of the telescopic OTA, and CMFB2 sets the reference voltage for the comparator by setting the current in the load branch. The resistors $R_{load}$ and $R_{Ref}$ in the load branch (Figure 10b) are sized such that the threshold voltage of the comparator is 40 mV. Referring to Figure 9, the DC voltage at the positive terminal of the comparator is set at 660 mV, and the negative terminal VC REF is set at 700 mV. Thus, the comparator is only triggered to output a high signal if the incoming voltage wave has an amplitude greater than or equal to 40 mV; otherwise, the output of the comparator is low. The 40-mV threshold was chosen so that amplified electronic noise from the CMUT device itself cannot trigger the comparator and produce false positives. Only a minimum detectable signal of 126 nA at the receiver input will be able to trigger the comparator. The minimum detectable signal is designed to be much larger than the noise floor to avoid false positives at the output. Since this is a pseudo-differential TIA, with one branch containing the negative feedback, only the positive terminal of the load branch contains the voltage signal, as shown in Figure 9, labeled $V_{OUT}$.

## 3. Results and Discussion

### 3.1. Analog Front End—Transceiver ASIC

**Figure 11:**
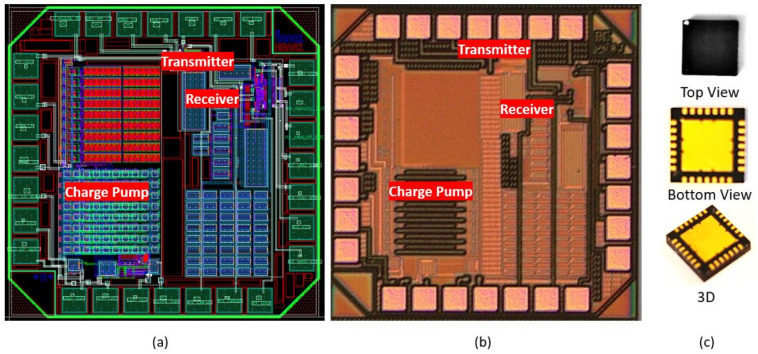
(**a**) ASIC layout; (**b**) fabricated ASIC; (**c**) QFN-28 packaged ASIC.

The circuit is designed and laid out in a 0.13 µm CMOS technology from IBM. The prototype chip occupies an area of 1 × 1 mm and contains the transmitter, oscillators, charge pump, and receiver as shown in Figure 11. The performance of the transmitter circuit is verified using pulses with an amplitude of 3.3 V as the input while ensuring the transmitter output signal is a clear pulse when driving a 108 pF load. At a pulse repetition rate of 156 Kpps and a pulse width of 730 ns, the current consumption of the transmitter circuit under these conditions is 17 µA. When the end voltage of 22 V has been reached, the high-frequency oscillator is turned off, and the low-frequency oscillator is used to maintain the voltage. The high-frequency oscillator is powered from a 1.2 V supply and oscillates at 19.5 MHz. After the charge-up period, the low-frequency oscillator is activated, and it generates a frequency of 26 kHz to maintain the voltage during the steady-state period. The charge pump circuit and HF oscillator consume power equivalent to 35.09 µW during the charge-up period and 439.2 nW during the steady-state period. This design results in significant power saving. The exact value of the charge pump’s ripple voltage is hard to determine due to the limited sensitivity of the scope (<20 mV).

It was simulated to be ~40 µV. The receiver circuit has a minimum detectable signal of 126 nA with a bandwidth of 7 MHz and a maximum total integrated noise current of 8.5 nA. Therefore, the input-referred noise current is 3.2$\frac{pA}{\sqrt{Hz}}$. The receiver circuit has a low power consumption of 72 µW. Table 1 summarizes the major performance metrics of the IC, and Table 2 compares this work with related works from the literature.

### 3.2. Micro-Viscometer System

**Figure 12:**
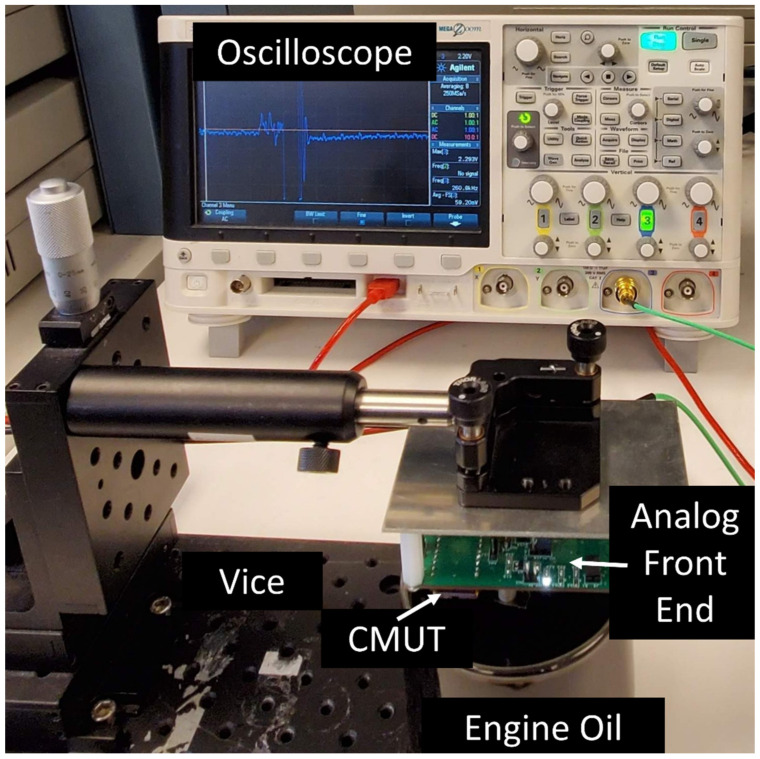
Test setup.

**Figure 13:**
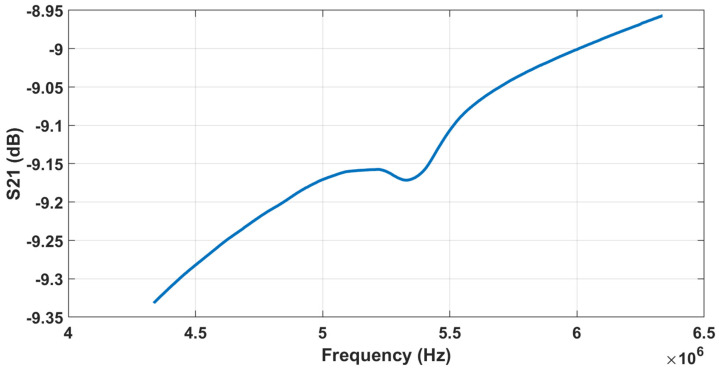
The measured resonance frequency of the CMUT when coated with Parylene-C.

The experimental setup depicted in Figure 12 was adopted to prove the functionality of the designed system. The setup involves the fabricated CMUT element, which has a membrane thickness of 1.5 µm and a radius of 60 µm. The measured resonance frequency of the CMUT when coated with Parylene-C is 5.3 MHz (Figure 13). The CMUT was immersed in engine oil to imitate an automotive oil tank and was actuated with an AC pulse signal (5 Vpp amplitude, 200 ns pulse width) while biased to emit ultrasound waves. The generated ultrasonic wave was transmitted through the engine oil and reflected from the bottom surface of the beaker. The acoustic signal was subsequently collected by the same CMUT. The final electrical signal is captured by an oscilloscope. The experiment was repeated for different engine oils with different viscosities: fresh 5W20, used 5W20, and fresh 15W40. The temperature of the engine oil in an automotive application was also taken into consideration. The oil was placed in a heated bath to homogenize the temperature of the engine oil in the beaker, and the tests were repeated at different temperatures (20.8, 40, 54, and 81.9 °C). In a parallel experiment, the viscosities of all the oil samples were measured using a Brookfield DV-E viscometer for reference.

**Figure 14:**
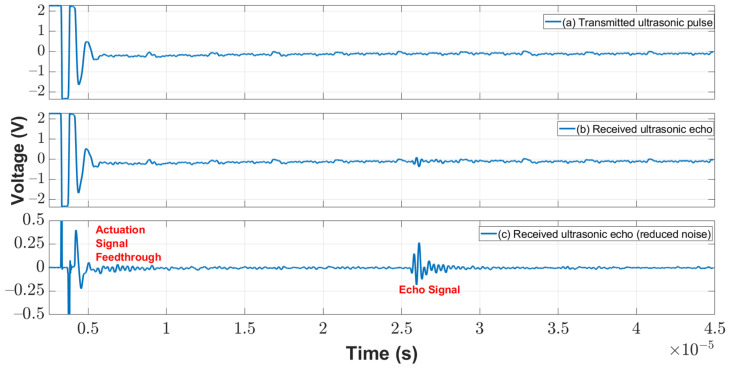
(**a**) Transmitted ultrasonic pulse, (**b**) received ultrasonic echo, and (**c**) the resultant subtraction is a reduced-noise version of the received echo.

The time-domain signal of the CMUT echo from the oscilloscope was converted to a frequency domain signal with the fast Fourier transform (FFT). Signal processing using FFT analysis to identify component frequencies was applied to capture the shift in the resonance frequency of the ultrasonic signal for different oil viscosities. The overall micro-viscometer system was tested to determine the frequency shift between fresh and used engine oil. First, the viscosity of the fresh 5W20 engine oil was measured using an industrial viscometer. Next, the CMUT signal was immersed in the fresh 5W20 engine oil. The transducer was then actuated to transmit an acoustic signal as shown in Figure 14a, and the same transducer received the echoed signal as shown in Figure 14b.

Spectral subtraction was applied to the signals to reduce the apparent noise as shown in Figure 14c. A Fourier transform was applied to the signal to obtain the resonance frequency of the signal when immersed in oil. This resonance frequency is compared to the fundamental resonance frequency of the CMUT (~5 MHz).

**Figure 15:**
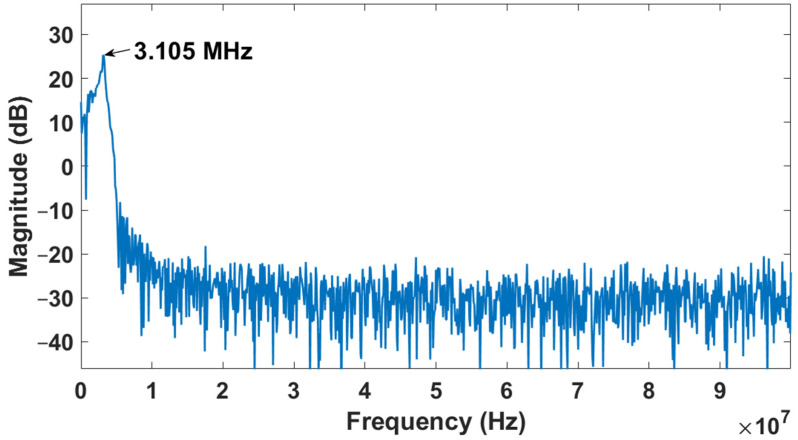
The resonance frequency of the CMUT immersed in fresh 5W20 engine oil.

**Figure 16:**
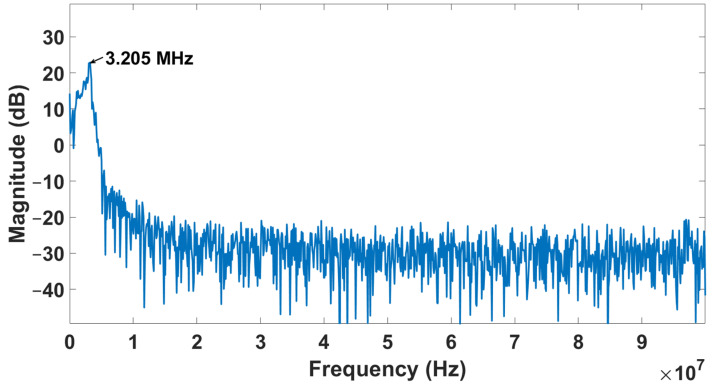
The resonance frequency of the CMUT immersed in used 5W20 engine oil.

The resonance frequency when the CMUT is immersed in the fresh 5W20 engine oil is shown in Figure 15. It is observed that the frequency has shifted from 5 to 3.105 MHz due to the loss in acoustic energy caused by the viscous damping of the engine oil and its radiation impedance. The same test was repeated under the same conditions on the used 5W20 engine oil. The measurements obtained using the proposed micro-viscometer system were validated using the reference Brookfield viscometer. The viscosity of the used 5W20 engine oil is 45.3 cP and is significantly lower than the viscosity of the fresh 5W20 engine oil (96.6 cP). This caused the frequency of the ultrasonic signal to shift to a higher value (3.205 MHz) in the used 5W20 engine oil, as shown in Figure 16.

**Figure 17:**
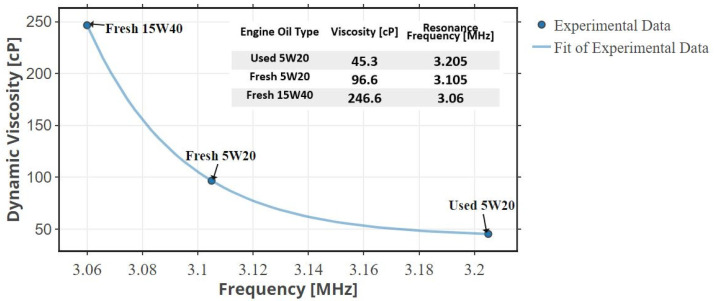
Resonance frequency comparison of different oil types (different viscosities) under the same conditions.

**Figure 18:**
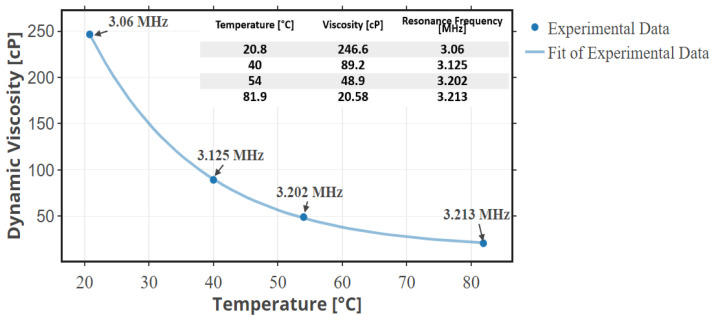
Resonance frequency comparison of an engine oil at varying temperatures.

Under the same conditions, the test was repeated on a highly viscous engine oil, 15W40. This oil has a viscosity of 246.6 cP at room temperature. The results of this test (Figure 17) demonstrate the sensitivity of the proposed CMUT based micro-viscometer’s resonance frequency to the viscosity of the fluid. When the CMUT is immersed in a higher viscosity fluid, the resonance frequency decreases. The new resonance frequency in the fresh 15W40 engine oil is 3.06 MHz. With reference to the 5W20 engine oil used for 3000 miles, the system has a sensitivity of 33 Hz/mile or −1.9 kHz/cP. Tests of the system in engine oil at different temperatures proved that, as temperature increases, the viscosity significantly decreases, and thus the resonance frequency increases as shown in Figure 18. Therefore, to utilize the sensor in an actual automotive setting, the sensor readings need to be taken when the vehicle’s motor is cool. Alternatively, a temperature sensor can be included in the micro-viscometer system to calibrate the effect of temperature on viscosity.

## 4. Conclusions

In summary, this paper reports the feasibility of a micro-viscometer for engine oil monitoring. The microsystem is based on a micro-machined ultrasonic transducer, namely a CMUT, and a low-power transceiver ASIC to form a compact solution. The system was tested on different engine oil types and at different temperatures. As the mileage usage increases, the viscosity decreases, and the resonant frequency of the proposed micro-viscometer system increases. As a result, this casts new light on the utilization of CMUT as an integrated sensor in the vehicle to monitor oil quality. The designed microsystem can lead to an optimally timed oil changing interval when the engine oil is no longer serviceable. This solution is efficient both economically and environmentally. It also provides an insight into the engine’s condition and can detect technical defects at an early stage to prevent engine damage. The proposed micro-viscometer paves the way for low-cost, small-size solutions for engine oil monitoring.

## Figures and Tables

**Table 1:** Measured performance metric of the fabricated IC.

Parameter	Value
Transmitter’s current consumption	9 µA (no load)
High oscillation frequency	19.5 MHz
Low oscillation frequency	25.67 kHz
Charge pump output voltage	22 V
Charge pump power consumption	439.2 nW during steady state (LF)
	35.09 µW during charge up (HF)
Receiver’s bandwidth	7 MHz
Receiver’s threshold	40 mV
Receiver’s power consumption	72 µW
Input-referred noise current	$3.2\frac{pA}{\sqrt{Hz}}$
Transimpedance gain	120.2 dBΩ
Receiver’s input impedance ^‡^	619.205 Ω

^‡^ Simulation results.

**Table 2:** Performance of the proposed receiver with previously reported designs.

Reference	Process (µm)	BW (MHz)	Power Consumption (mW)	Gain (dBΩ)	Input Resistance (kΩ)	Input Referred Noise Current $\left(\frac{pA}{\sqrt{Hz}}\right)$
[25]	0.065	10	150	107	2.15	0.28
[26]	0.8	9–11	3	72–90	1	-
[27]	0.18	75	0.48	61.18	310	16.8
[28]	0.18	2.76	11	65	-	5.6 × 10^4^
[29]	0.065	7.5	0.18	79–97	-	4.8
This Work	0.13	7	0.072	120.2	0.6192	3.2

## Data Availability

The data that support the findings of this study are available from the corresponding author upon reasonable request.
